# Management of chronic spontaneous urticaria (CSU): a treat to target approach using a patient reported outcome

**DOI:** 10.1186/s13223-017-0210-0

**Published:** 2017-08-24

**Authors:** Hermenio Lima, Melinda Gooderham, Jan Dutz, Charles Lynde, Hugo Chapdelaine, Anne Ellis, Martin Gilbert, Vincent Ho, Kim Papp, Yves Poulin, Gordon Sussman

**Affiliations:** 1 Division of Dermatology, Department of Medicine, McMaster University, HSC-3U8, 1280 Main St. West, Hamilton, ON L8S 4K1 Canada; 2 SKiN Centre for Dermatology and Probity Medical Research, Peterborough, ON Canada; 3 Department of Dermatology and Skin Science, University of British Columbia, Vancouver, BC Canada; 4 Lynde Institute for Dermatology, Markham, ON Canada; 5 Division of Allergy & Immunology, Department of Medicine, Université de Montréal, Montreal, QC Canada; 6 Division of Allergy & Immunology, Department of Medicine, Queen’s University, Kingston, ON Canada; 7 Hôpital du Saint-Sacrement du CHU de Québec, Quebec City, QC Canada; 8 Clinical Research and Probity Medical Research, Waterloo, ON Canada; 9 Department of dermatology, Hôpital Hôtel-Dieu du CHU de Québec, and Centre de Recherche Dermatologique du Québec métropolitain, Quebec City, QC Canada; 10 Division of Allergy and Clinical Immunology, St. Michael’s Hospital, Toronto, ON Canada

**Keywords:** Chronic spontaneous urticaria, Chronic idiopathic urticaria, Urticaria, Urticaria Activity Score, CSU, CIU, UAS

## Abstract

**Background:**

Treat-to-target therapy approaches are established for chronic diseases such as diabetes, hypertension, and more recently rheumatoid arthritis, resulting in improved patient outcomes. These approaches do not use patient reported outcomes (PRO) as targets of therapy. Chronic spontaneous urticaria (CSU), also called chronic idiopathic urticaria (CIU), is defined as recurrent urticaria of known and unknown cause, lasting more than 6 weeks. Treatment of CSU can be challenging. However, with the advent of proven therapies and validated instruments for measuring disease activity, the concept of treat-to-target (T2T) can be successfully applied to CSU. Herein, we propose a potential PRO therapeutic target and suggest a T2T approach for the management of patients with CSU.

**Methods:**

Principles and recommendations for a treat-to-target approach in CSU (T2T/CSU) were developed by a Canadian task force, consisting of dermatologists, immunologists, and allergists. The task force formulated recommendations for therapeutic targets in CSU on the basis of a systematic literature review and expert opinion.

**Results:**

The key features of these T2T/CSU recommendations are the use of a PRO as the principal target, with symptom control as measured by Urticaria Activity Score 7 (UAS7 ≤ 6), targeting symptom remission (UAS7 = 0).

**Conclusion:**

Treatment targets such as UAS7 ≤ 6 and UAS7 = 0 provide a benchmark for success in the care of patients with CSU, and will permit the evaluation of a PRO-based T2T approach in the care of these patients and the effect of this approach on improved patient care as seen in other chronic diseases.

## Introduction

Optimal treatment of chronic diseases requires tight control of disease activity beyond improvement of patients’ symptoms. Treat-to-target (T2T) treatment strategy aims to achieve complete symptom control and improve the patient’s quality of life (QoL). When compared to the traditional symptom-based approach, target-based strategies have been shown to lead to better treatment and patient outcomes in diseases with complex clinical presentations and a paucity of overt symptoms [1]. T2T has been established for chronic diseases in which clinical severity correlates with biomarkers or aggregate measurements of disease activity [2]. Designing a treatment target requires tight control of disease activity based on values of specific quantitative measures [3]. T2T approach in hypertension, diabetes, and rheumatoid arthritis (RA) has led to a reduction of organ damage, improvement in patient’s QoL and decrease in mortality rates [4]. Such an approach has been less common in dermatologic disease.

Chronic spontaneous urticaria (CSU); a mast-cell driven disease, characterized by recurrent itchy wheals (hives), is defined as spontaneous appearance of wheals, angioedema, or both, for 6 or more weeks, with no apparent external trigger [5, 6]. Common therapeutic goals are reduction in disease activity, complete symptom control, and improvement in QoL. CSU is a major burden to patients with significant healthcare costs and socio-economic implications [7, 8].

Chronic spontaneous urticaria is similar in many aspects to chronic diseases for which a T2T approach has been established. A validated clinical activity tool, the Urticaria Activity Score 7 (UAS7), allows clinicians to monitor levels of disease activity in response to treatment in CSU and may thus be used in a treat to target approach. UAS7 is a composite measure of clinical findings similar to composite measures used to quantitate clinical activity in diabetes, hypertension, coronary heart disease and rheumatoid arthritis that use a single biomarker to define treatment targets. However, unlike other markers, UAS7 is a patient-reported outcome (PRO) measure used in clinical trials evaluating the efficacy of therapy in CSU [9]. UAS7 is a validated tool that measures levels of disease activity, and includes two items—intensity of pruritus (itch) and number of wheals (hives) [10].

While CSU differs from a few other chronic diseases by the absence of long-term organ damage and low mortality rates, a T2T approach offers an important management tool guiding therapeutic modification until symptom control or remission is obtained. The task force formulated three overarching principles and five recommendations for therapeutic targets in CSU on the basis of a systematic literature review and expert opinion. The key component of these recommendations is to achieve complete symptom control and improve the QoL. The Canadian T2T in CSU recommendations is meant to inform physicians about therapeutic targets as a management tool used to help patients reach optimal outcomes in the treatment of CSU. The recommendations are in line with the Canadian and EAACI/GA2LEN/EDF/WAO urticaria clinical practice guidelines [11, 12].

## Methods

A Steering Committee (SC) involving allergists, immunologists and dermatologists was assembled creating a Canadian task force to developed the principles and recommendations for a treat-to-target approach in CSU in November 2014. SC members were identified by their proficiency in managing CSU; participation in consensus reports; involvement in clinical trials for CSU; and relevant medical activity in different regions of Canada.

After reviewing the published literature in CSU, and deliberating upon the treatment targets for this paper, the SC panel formulated a provisional set of recommendations in line with the Canadian and EAACI/GA2LEN/EDF/WAO urticaria clinical practice guidelines [13, 14].

Provisional recommendations were presented for discussion, amendment and voting by SC members. SurveyMonkey^®^ instrument was used by SC members to submit information. Participants voted on each recommendation statement. Statements receiving ≥75% of votes were approved while those with ≤25% were rejected. Any statements receiving >25 and <75% of votes were subjected to an additional debate, and only those receiving ≥67% support were accepted. Therefore, a majority of ≥50% support was required for a statement to be accepted. For each accepted statement, the group was required to express their level of agreement using a 5-point numerical rating scale (1 = do not agree to 5 = agree completely) and the level of agreement was expressed as the average of all voters.

These T2T recommendations were discussed, amended and voted upon by Task Force members. In a final step, the statements were distributed by email to each Task Force participant for comments. Only modifications of syntax and improved clarity were considered. Changes to meaning were not accepted.

## Results

The online SurveyMonkey^®^ results are presented in Table 1. The overarching principles and recommendations for the T2T/CSU are discussed in detail below.

**Table 1:** Overarching principles and recommendations results of the SurveyMonkey^®^ online responses

	Support (%)	Agreement^a^
Overarching principles		
Patient and physician must share the responsibility of treatment for CSU/CIU	100	4.9
Achieve symptom control and normalize patient’s long-term health-related quality of life	100	4.4
A record of UAS7 must be part of any urticaria treatment to target management plan by measuring disease activity and adjusting therapy accordingly	100	4.6
Recommendations		
The primary target for treatment of CSU/CIU should be clinical symptom remission	100	4.4
The patient should be knowledgeable about the treatment target under physician supervision	100	4.8
The use of a validated tool to monitor disease activity	100	4.6
Until the treatment target is reached, drug therapy should be adjusted	77.8	3.7
The treatment target should be sustained throughout the remaining course of the disease management	100	4.6

^a^The level of agreement was expressed as the average of all voters on a 5-point numerical rating scale. A majority of ≥50% was required for a statement to be accepted

### Overarching principles

#### Patient and physician must share the responsibility of treatment for CSU

Management of CSU must occur in a close collaboration between the patient and physician [15]. The patient must be involved in symptom evaluation as the key metric for success in a PRO measure (the UAS7). The patient must also be involved in the determination of treatment goals. The physician must inform the patient of all available treatment options, potential complications of therapy, and the rationale for recommending a particular therapeutic approach based on patient’s disease severity.

#### Achieve symptom control and normalize patient’s long-term health-related quality of life

The therapeutic objective in CSU is to attain symptom control and symptom remission, while minimizing treatment-related side effects to ultimately improve quality of life (QoL). Symptom control and symptom remission are based on both patient’s feedback (PRO—using the UAS7) and physical examination. While symptom remission should be a clear target (UAS7 = 0), based on analysis of the minimal clinically important difference in UAS7 scores, low disease activity (UAS7 ≤ 6) may be an acceptable alternative therapeutic goal [16, 17]. In addition, use of the validated chronic urticaria quality of life questionnaire (CU-Q2oL) to assess the effect of CSU on QoL is recommended to monitor aspects of QoL such as anxiety and stress that are not captured by the UAS7 [11, 18, 19].

#### Record of UAS7 must be part of any urticaria treatment to target management plan for measuring disease activity and adjusting therapy accordingly

Management of CSU is aimed primarily at symptom control while minimizing treatment-related side effects. Recent guidelines recommend a step-wise pharmacological approach to the management of CSU with assessment of disease activity and QoL at each stage [13]. In a T2T/CSU approach, a record of UAS7 could be used as an indicator of disease activity, and as a tool to guide treatment decisions and to modify therapy based on response to treatment with a goal of complete symptom control (UAS7 ≤ 6) or symptom remission (UAS7 = 0) [16].

### Recommendation statements

#### The primary target for treatment of CSU should be clinical remission

##### Symptom remission is a critical goal for all patients

Clinical symptom remission is defined as complete symptom control and the lack of significant disease activity [11]. Few studies have reported symptom remission as the clinical trial primary endpoint [20]. The majority of the trials have evaluated the frequencies of symptom remission in response to different therapies, or had symptom remission as the primary endpoint, but this was investigated by static management and not by strategic therapy escalation as in a treat to target approach [21–24].

##### A significant proportion of patients can achieve symptom remission

A meaningful proportion of patients can attain sustained symptom remission, and avoid the morbidity associated with CSU, when treatment is initiated early in the course of the disease. Therefore, symptom remission (UAS7 = 0) should be a meaningful and attainable target for all patients with CSU [25].

##### Significant residual disease activity is not acceptable

Only a few studies have dealt with the natural course of CSU, which can be active for as long as 20 years in the absence of therapy despite a tendency of spontaneous recovery [26]. Any level of disease activity (UAS7 > 6) may contribute to recurrence of acute flare-ups. The aim of therapy for CSU is to achieve symptom control (UAS7 ≤ 6) and sustained remission (UAS7 = 0), with the ultimate goal of improving patient’s QoL [27].

#### The patient should be knowledgeable about the treatment target under physician supervision

##### Importance of discussing the therapeutic options, treatment strategies, and the reason for selected treatment target with the patient

T2T approach includes patient involvement in shared decision-making [28]. Minimization of symptoms is a target of treatment in CSU and can be monitored using measures of disease activity such as UAS7, and Urticaria Control Test (UCT). However, due to its recent introduction, the UCT has not yet been widely used [29, 30].

##### The role of the physician is to define the treatment target with the patient, choose the appropriate therapy and follow the patient over time to monitor disease activity

Physicians following the T2T principles should help the patient to understand the goal of the treatment of CSU. A patient-friendly version of T2T/CSU including UAS7, the risks and benefits of treatment, and control of disease activity should be provided to start the dialogue [31]. Studies have shown that even in a busy clinic it is possible to create a target for treatment with a patient, expressed as an activity to be retained or regained [8, 32].

#### The use of a validated tool to monitor disease activity

##### Assessment of CSU by using the most appropriate instrument is needed in routine clinical practice to guide treatment decisions. We recommend the UAS7 score for monitoring response to therapy

The UAS7 is a validated tool with which to follow CSU disease activity in clinical practice [33]. Continued measurement of disease activity is needed in routine clinical practice to guide treatment decisions. The UAS has proven to be as useful as other complex symptom scores. Patients score both their pruritus and number of wheals daily on a three-point scale, which gives them a daily score out of 6. The sum of the daily UAS scores gives the score for that week, with a maximal score of 42. For symptom control the target should be UAS7 ≤ 6, and for remission, UAS7 = 0. Despite its limitations, this scoring system gives the clinicians and patients a quantitative method of monitoring disease activity and response to treatment [29].

##### Disease activity must be regularly measured and monitored

Pruritus and wheals are manifestations of inflammation in CSU [8]. Inflammation can be monitored by a regular assessment of disease activity using UAS7. To help control disease activity, one suggestion is to follow patients with UAS7 ≥ 6 as frequently as monthly, and less frequently (e.g. every 3–6 months) for patients with sustained symptom control (UAS7 ≤ 6) or remission (UAS7 = 0) [34].

##### The target value of disease activity level may be influenced by comorbidities

Other conditions should be considered when making clinical decisions, in addition to assessing composite measures of disease activity. A target value of UAS7 may have to be less stringent in patients with comorbidities (e.g. chronic infections, renal or hepatic functional impairment, chronic congestive heart failure, anxiety or depression, etc.) or associated therapies. The practice parameters developed by the joint task force on practice parameters (JTFPP), include omalizumab as a fourth-line treatment option, stressing that the benefits need to be weighed against the potential for burden and cost [35, 36]. However, omalizumab is relevant in all CSU/CIU guidelines [37].

#### Until the treatment target is reached, drug therapy should be adjusted using a ladder approach according to current AACI/GA2LEN/EDF/WAO therapy guidelines

##### Dose adjustment of existing medication

A change to the treatment plan should be made for patients who have not achieved the well-controlled primary target of symptom control (UAS7 ≤ 6) or remission (UAS7 = 0). The type of adjustment depends on the applied strategy and patient’s individual response. However, a change in drug therapy is not always necessary. For patients who have not achieved the primary target of remission but show significant improvement over the previous 2 weeks, dose adjustment or continuation for another 2 weeks instead of changing therapy may be sufficient.

##### Assessment follow-up using T2T—utility of UAS7

The effect of treatment on symptoms of CSU should be measured based on the UAS scores. While the UAS scores is a patient-reported outcome measure, physician evaluations are independent of the opinion of the patient. Thus, the process by which the physician and the patient make a decision together for therapeutic modification is based on a dialogue, builds on the preferences of the patient, and the knowledge of the doctor. While remission is a desired treatment target, low disease activity and symptom control (UAS7 ≤ 6) may be an acceptable alternative therapeutic goal [38].

However, until the desired treatment target is reached, therapy should be adjusted at least biweekly [39]. UAS7 > 6 warrants frequent follow-up and assessment of the disease status in order to adjust or modify treatment accordingly. If symptom control (UAS7 ≤ 6) or remission (UAS7 = 0) is reached and sustained, fewer evaluations are acceptable. If UAS7 ≤ 6 is not reached within 2–4 weeks from starting or adjusting therapy, treatment should be modified.

#### The treatment target should be sustained throughout the remaining course of the disease management

##### Maintaining the state of symptom control or remission continuously

Once CSU disease activity has been titrated to a target of symptom control or remission, this state should be maintained continuously.

##### Further studies are needed to help direct decisions to reduce or discontinue treatment for CSU

Studies evaluating discontinuation of therapy once remission is reached, patient follow-ups to monitor flare-ups, and re-initiation of therapy, are scarce [39]. Furthermore, few studies address the frequency of relapses with a particular therapy or duration of treatment [40, 41]. One approach, particularly for patients with long-standing CSU, is to manage patients who have achieved remission with a specific therapeutic agent for an additional 3 months before tapering the dosage [42].

## Discussion

Treating-to-target, as in other chronic diseases, should be the goal of CSU management. Such a T2T strategy has been shown to lead to better patient outcomes when clinically relevant targets such as biomarkers of disease activity, and composite clinical disease activity measures are used. While guidelines for treating CSU have been established, none discuss a treatment to target approach to achieve the therapeutic goal of symptom remission or disease control. These principles and recommendations for a treat-to-target approach were based on the best treatment outcome of CSU in a busy clinical practice. These T2T/CSU recommendations, however, are based on a literature review and expert opinion of the current treatment guidelines, and they evolved from discussions among experts from across Canada. The requirement of a high level of agreement from the expert opinion on most of the statements implies a broad acceptance and consensus.

Treatment strategies improve compliance and achieve a better result when the patient has access to therapeutic strategies suggested in guidelines. However, a Canadian task force was aware of potential financial constraints or access to particular treatments, which would only allow a certain proportion of patients to achieve the desired targets. These T2T guidelines differ from those of other chronic disease in which biomarkers or physician determined composite disease activity scores are commonly used to establish therapeutic targets [1]. These guidelines also differ from recent guidelines for the treatment of psoriasis where physician-assessed disease activity is combined with a PRO score, the DLQI [43]. The nature of CSU symptoms and intermittent clinical findings, lends itself best to a T2T guideline relying on a PRO, the UAS7, with the possible adjunct use of an additional QoL questionnaire, the CU-Q2ol (Table 2).

**Table 2:** Treat-to-target strategies for chronic diseases [1, 39]

Disease	Diabetes	Hypertension	Rheumatoid arthritis	Psoriasis	CSU
Target	HbA1c	BP level	DAS28	PASI + PROQOL	UAS7^a^
Comment	Biologic marker	Biologic marker	Clinical score	Clinical score Patient input	Patient input

*BP* blood pressure, *CSU* chronic spontaneous urticaria, *DAS28* disease activity score-28, *HbA1c* hemoglobin A1c, *PASI* psoriasis area severity index, *PROQOL* patient reported outcomes quality of life, *UAS7* weekly urticaria activity score

^a^Recommendation from this publication

These recommendations are meant to provide guidance towards therapeutic goals in CSU, enabling iterative physician evaluation and treatment modification, based upon a PRO. We propose that bringing them into practice will help enable symptom control and normalization of patient’s QoL. The utility and efficacy of a T2T strategy using a PRO as prime indicator of success will need to be evaluated in formal studies, but promises to conceptually extend the framework for T2T approaches to treatment.

The UAS7, a validated, clinically relevant, and readily applied PRO measurement tool, can be used to define a treatment target in CSU. We propose here that a relevant PRO can be used in defining a T2T strategy for CSU. An appropriate and achievable treatment target is symptom control (UAS7 ≤ 6) or symptom remission (UAS7 = 0). Successful management of CSU is to achieve and maintain this target.
